# Role of cooperative factors in the photocatalytic activity of Ba and Mn doped BiFeO_3_ nanoparticles†

**DOI:** 10.1039/d1na00420d

**Published:** 2021-08-26

**Authors:** Astita Dubey, Alexander Schmitz, Vladimir V. Shvartsman, Gerd Bacher, Doru C. Lupascu, Marianela Escobar Castillo

**Affiliations:** Institute for Materials Science and Center for Nanointegration Duisburg-Essen (CENIDE), University of Duisburg-Essen 45141 Essen Germany astita.dubey@uni-due.de; Werkstoffe der Elektrotechnik & CENIDE, Universität Duisburg-Essen Bismarckstraße 81 47057 Duisburg Germany

## Abstract

The escalated photocatalytic (PC) efficiency of the visible light absorber Ba-doped BiFe_0.95_Mn_0.05_O_3_ (BFM) nanoparticles (NPs) as compared to BiFeO_3_ (BFO) NPs is reported for the degradation of the organic pollutants rhodamine B and methyl orange. 1 mol% Ba-doped-BFM NPs degrade both dyes within 60 and 25 minutes under UV + visible illumination, respectively. The Ba and Mn co-doping up to 5 mol% in BFO NPs increases the specific surface area, energy of d–d transitions, and PC efficiency of the BFO NPs. The maximum PC efficiency found in 1 mol% Ba doped BFM NPs is attributed to a cooperative effect of factors like its increased light absorption ability, large surface area, active surface, reduced recombination of charge carriers, and spontaneous polarization to induce charge carrier separation. The 1 mol% Ba and 5 mol% Mn co-incorporation is found to be the optimum dopant concentration for photocatalytic applications. These properties of co-doped BFO NPs can, *e.g.*, be exploited in the field of water splitting.

## Abstract 2

The escalated photocatalytic (PC) efficiency of the visible light absorber Ba-doped BiFe_0.95_Mn_0.05_O_3_ (BFM) nanoparticles (NPs) as compared to BiFeO_3_ (BFO) NPs is reported for the degradation of the organic pollutants rhodamine B and methyl orange.

## Graphical abstract

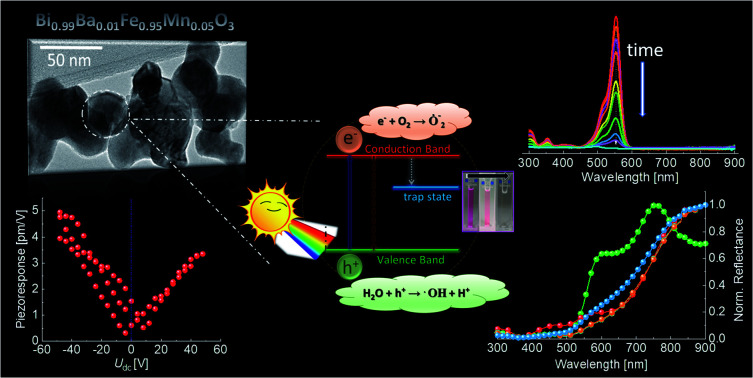

## Introduction

The recycling of wastewater contaminated by organic pollutants is one of the major priorities in our ecosystem. In this area, research is getting promoted on processes that improve the oxidative degradation of organic pollutants. These processes include photocatalysis,^1^ Fenton oxidation,^2^ and ozonation.^3^ In the advanced oxidation processes like heterogeneous photocatalysis, water purification happens on the surface of a photocatalyst under the irradiation of photons resulting in total mineralization of the dyes. The efficiency of the method is based on the formation of the hydroxyl radical ·OH, which acts as an oxidation agent and is responsible for the photodegradation of the organic pollutants.

In the field of photocatalysis, semiconductors have contributed immensely.^4^ The state-of-the-art photocatalytic materials like TiO_2_, ZnO *et al.* can only degrade the pollutants under UV light, which excludes the visible light range of the solar spectrum. Their application as antibacterial and photoelectrochemical agents are quite promising, like *e.g.*, for ZnO and TiO_2_ respectively.^5,6^ Presently, there is a demand for photocatalysts, which can not only utilise a maximum range of solar light, but are a low cost and sustainable technology for the wastewater treatment.^7,8^ Single phase multiferroic BiFeO_3_ (BFO),^9^ is a p-type semiconductor,^10^ and features a narrow band gap, non-toxicity and chemical inertness making it a promising candidate for photocatalytic applications.^11,12^

BFO and doped BFO nanoparticles (NPs) are ferroelectric materials.^13,14^ It has been shown that in photoferroelectric materials^15^ the spatial separation of electrons (e^−^) and holes (h^+^) and their transfer from bulk to the surface is assisted by the presence of intrinsic spontaneous polarization induce local electric fields known as depolarization fields.^16^ The role of this intrinsic electric field for charge carrier dynamics in the ferroelectric photocatalyst for the photocatalytic redox reactions is fascinating, but the mechanisms are still not understood. After poling, a significant enhancement in the visible-light photocatalytic activity of BFO NPs was reported recently.^17^ BFO has been studied for the decomposition of organic dyes under UV + visible light irradiation.^18^ However, the utilisation of BFO is limited due to rapid charge carrier recombination, and the formation of detrimental defect and trap states in the electronic band structure due to oxygen vacancies. Subsequently, different strategies have been developed to improve its photocatalytic efficiency, *e.g.*, by altering the morphology,^19–21^ synthesizing composites,^22–25^ exploring nanohybrids,^26^ and tailoring its properties *via* doping.^27^

A moderate amount of doping into BFO at both Bi- and Fe-sites affects its ferroelectric, magnetic and optical properties.^14,28^ It has been shown that cation doping can reduce the particle size and alter the band gap of pristine BFO.^29^ These fruitful changes increase the surface to volume ratio and enhance the visible light absorption of the NPs, which are important parameters for photocatalytic application. In this context, rare-earth metal doping has been widely studied to improve the photocatalytic properties of BFO. Rhodamine B (RhB) dye was degraded using Gd-doped BFO NPs.^30^ The exploitation of Gd and Sn co-doped BFO NPs and La and Se co-doped BFO NPs was reported for the photodegradation of several dyes like methylene blue (MB), congo-red (CR) and methyl violet (MV).^31,32^ Like this, there are many other reports on Nd, Dy, La, and Sm-doped BFO materials based on complicated synthesis methods.^33–36^ From an economic and environmental point of view rare-earth metal doping is not justifiable, especially for water cleaning purposes.

Studies on photodegradation of dyes by alkaline and transition metal doped BFO NPs are also found to be effective. Single doped BFO NPs like, Sc-doped BFO NPs and nanofibers were utilised for the photodegradation of MB dye.^37^ Mn-doped BFO NPs were able to degrade acid red dye, and Ba doped BFO NPs was effectual to degrade MO dye.^38,39^ The photodegradation of CR dye using Ba and Mn co-doped and Ca doped BFO nanofibers was reported recently.^40,41^ In spite of its calibre in photocatalysis, nanofiber production is difficult and not worthwhile for large-scale application. To overcome this, an economical synthesis route is required with low-cost dopants without compromising the advantageous properties of BFO NPs. Ba and Mn as dopants are inexpensive and amply available elements on earth. It has been shown that these elements are easy to incorporate, and their doping bestows the properties of BFO.^14,42^ The versatile oxidation state and similar ionic radius of Mn makes it one of the best transition metals to substitute Fe.

Nevertheless, to our knowledge there is no study on Ba and Mn co-doped BFO NPs for photocatalysis. To obtain such NPs we have implemented a modified sol–gel method, which is an easy, cost-effective, and quite reproducible route. We have incorporated from 1 to 5 mol% of Ba into BiFe_0.95_Mn_0.05_O_3_ (BFM) NPs. We study the role of these dopants in the overall increase of photocatalytic efficiency of the BFO NPs. There are several factors which affect the rate of degradation of dyes including surface area, light absorption, charge carrier separation, and their recombination rate [Fig. S1†]. We find that 1 mol% Ba doped BFM NPs show the best photocatalytic activity among all co-doped NPs examined in this study and can completely degrade MO and RhB dyes within 25 and 60 minutes, respectively, for 10^−5^ M dye concentration. We found that an optimum dopant concentration is the key factor to control the photocatalytic parameters.

## Experimental

### Sample preparation

Ba and Mn co-doped BFO NPs (Bi_1−*x*_Ba_*x*_Fe_0.95_Mn_0.05_O_3_: *x* = 0.00, 0.01, 0.03, 0.05) were synthesized by a modified wet chemical ‘sol–gel route’ assisted by a calcination step at 773 K.^14^ Bismuth nitrate Bi(NO_3_)_3_·5H_2_O (≥98%), iron nitrate Fe(NO_3_)_3_·9H_2_O (≥98%), manganese acetate Mn(CH_3_COO)_2_·4H_2_O (≥99%), barium hydroxide Ba(OH)_2_·H_2_O (99.995%), tartaric acid C_4_H_6_O_6_ (≥99%), and nitric acid (HNO_3_) were purchased from Sigma Aldrich and were used as precursors without further modification. We use the following abbreviations in the manuscript BiFe_0.95_Mn_0.05_O_3_ = BFM, Bi_0.99_Ba_0.01_Fe_0.95_Mn_0.05_O_3_ = 1BBFM, Bi_0.97_Ba_0.03_Fe_0.95_Mn_0.05_O_3_ = 3BBFM, Bi_0.95_Ba_0.05_Fe_0.95_Mn_0.05_O_3_ = 5BBFM.

### Photocatalysis (PC) procedure

10^−5^ M of methyl orange (MO) and rhodamine B (RhB) aqueous suspensions were prepared. To establish an adsorption–desorption equilibrium between the dye and the photocatalyst, 50 ml of organic dye with 0.025 g of NPs were placed into a jacketed beaker in the dark for 30 minutes. To achieve good dispersion of the ligand free NPs in the dye solution, 9 minutes ultrasonication was performed in the dark. The degradation of RhB and MO was carried out under the irradiation of a halogen lamp (LOT-Oriel) in a dark chamber to avoid additional light sources. During PC experiments, continuous magnetic stirring of the NPs was performed to avoid their settling. A UV cut-off filter (390 nm cut-off wavelength) was used for the experiments under visible light only.

For the PC in acidic medium, the pH of the solution (dye + NPs) was adjusted up to 2.2 by adding a few drops of 2 N HNO_3_. To keep a constant temperature (298 K) during PC, a continuous water flow was maintained around the jacketed beaker to rule out any thermal catalytic effect. To study the degradation of the dye, 1 ml solution was taken every 5 minutes and centrifuged using a Hettich Zentrifugen Universal 320 R device at 9000 RPM for 10 minutes in the dark to separate the NPs from the dye. Absorption measurements were then performed on the obtained solution.

After the complete degradation of the dye (colourless solution), the NPs were conveniently separated with the help of magnets under the PC vessel and used for the next cycle of PC. After 3 successful PC cycles, the NPs were again structurally characterized to compare with previous results.

## Characterization

### X-ray diffraction (XRD)

The phase and lattice parameters of all NPs were investigated by powder XRD using a Panalytical Empyrean (Cu K_α_ radiation) diffractometer over a 2-theta range of 10° to 80° with a step size of 0.026°. The lattice parameters were obtained by Rietveld refinement using the High Score Plus software with consideration of nonstructural effects, crystallite size, and strain.

### Scanning electron microscopy (SEM)

An ESEM Quanta 400 FEG, FEI instrument was used to study the shape and morphology of the NPs. The atomic composition of the NPs was estimated by energy dispersive X-ray spectroscopy (EDXS) using an analytical SEM device (EDS, energy resolution <132 eV for Mn Kα, detector area 10 mm^2^).

### Transmission electron microscopy (TEM)

The size and crystallinity of the NPs were analyzed using a high-resolution TEM on a JEOL JEM-2200FS microscope using 200 kV acceleration voltage and a probe side aberration corrector.

### Nitrogen physisorption measurements

The specific surface area of the NPs was determined from nitrogen sorption measurements using a Coulter SA 3100 analyzer (Beckman Coulter). The samples were degassed under vacuum at room temperature for 24 h. The Brunauer–Emmett–Teller (BET) equation was applied to determine the specific surface areas of the NPs. The Barrett–Joyner–Halenda (BJH) method was applied to determine the pore volume distribution.

### Piezoresponse force microscopy (PFM)

To address the ferroelectric properties of the NPs, PFM measurements were performed using a commercial scanning probe microscope MFP-3D (Asylum Research). Pt/Cr coated cantilevers Multi 75E-G (Budget Sensors) with a spring constant of 3 N m^−1^ were used. PFM measurements were conducted at a probing voltage of amplitude *U*_ac_ = 5 V and frequency *f* = 50 kHz. The NPs were drop-cast onto conductive carbon tape for the PFM measurements.

### UV-Vis spectrometry

The diffuse reflectance spectra (DRS) of the NPs were collected by a UV-Vis spectrometer Shimadzu 2600 in the wavelength range 300–900 nm. The NP-powder was pressed and then measured inside an integrating sphere in reflectance mode. Barium sulfate was used as a reference and the baseline was corrected before recording each spectrum. Transmission spectra of diluted NPs and NPs drop-cast onto quartz substrates were recorded using a Shimadzu UV-2550 spectrometer equipped with an integrating sphere in transmission geometry.

### Fluorescence spectrometry

To measure the emission spectra, a Varian Cary Eclipse Fluorescence spectrophotometer was used. The 450 nm laser excitation source was used to collect emission spectra in the range from 550–650 nm. A stable dispersion of NPs was prepared in ethanol (optical grade) and a baseline was obtained by measuring pure ethanol.

### Photoluminescence (PL) spectroscopy

Temperature-resolved PL measurements were conducted inside a CryoMech PT-403 closed-cycle cryostat under high vacuum conditions (<10^−5^ mbar). The samples were excited with the filtered 325 nm line of a Kimmon IK-series He–Cd laser. Luminescence spectra were collected using a fused-silica lens system and dispersed using a Horiba iHR320 grating monochromator before being recorded by a Horiba Symphony I CCD camera. Samples were dissolved in optical grade ethanol and treated in an ultrasonic bath for 30 minutes. 20 μL of the dispersion were then drop-cast onto a cleaned Si substrate and dried at room temperature. A reference sample was prepared by drop-casting 20 μL of ethanol onto an identical Si substrate.

## Results and discussion

### Structure and morphology of doped BFO NPs

**Fig. 1:**
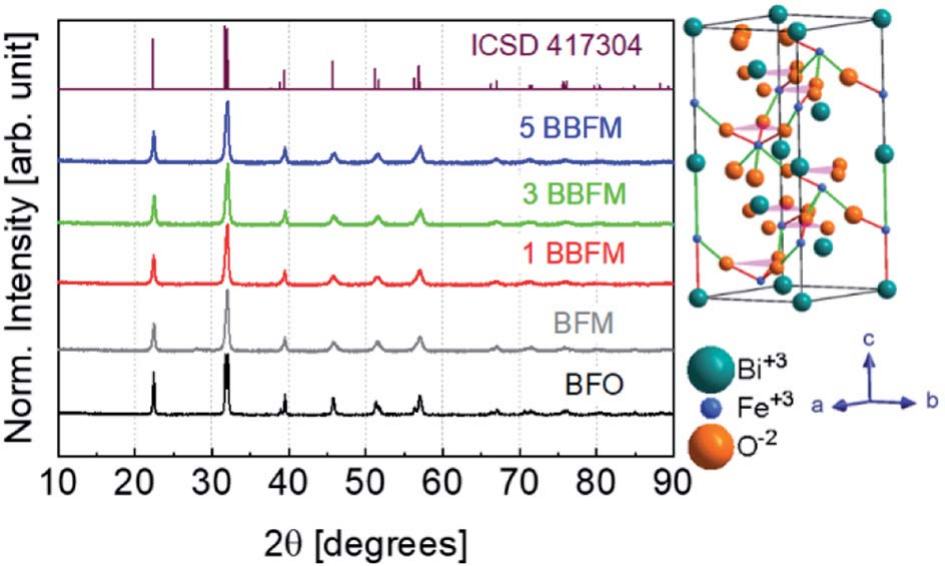
XRD diffractograms of BFO, BFM, 1BBFM, 3BBFM, and 5BBFM NPs along with their rhombohedral (*R*3*cH*) crystal structure on the right.

**Fig. 2:**
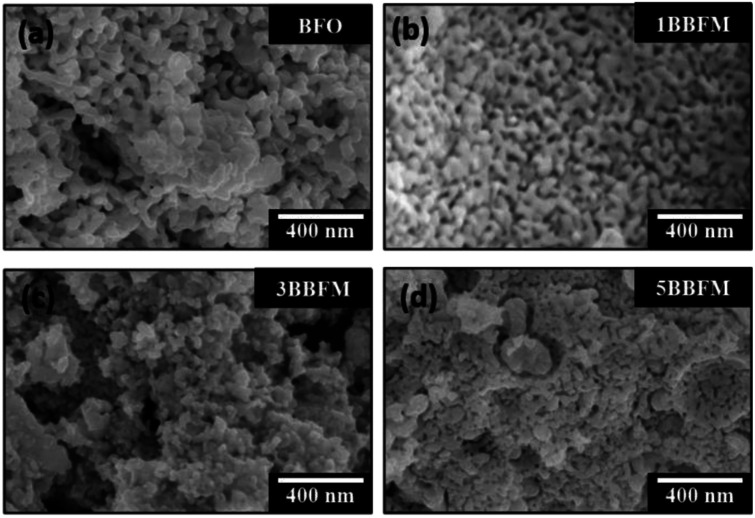
SEM images of undoped (a) and Ba and Mn co-doped BFO NPs with Ba concentrations of 1 mol% (b), 3 mol% (c), and 5 mol% (d).

**Fig. 3:**
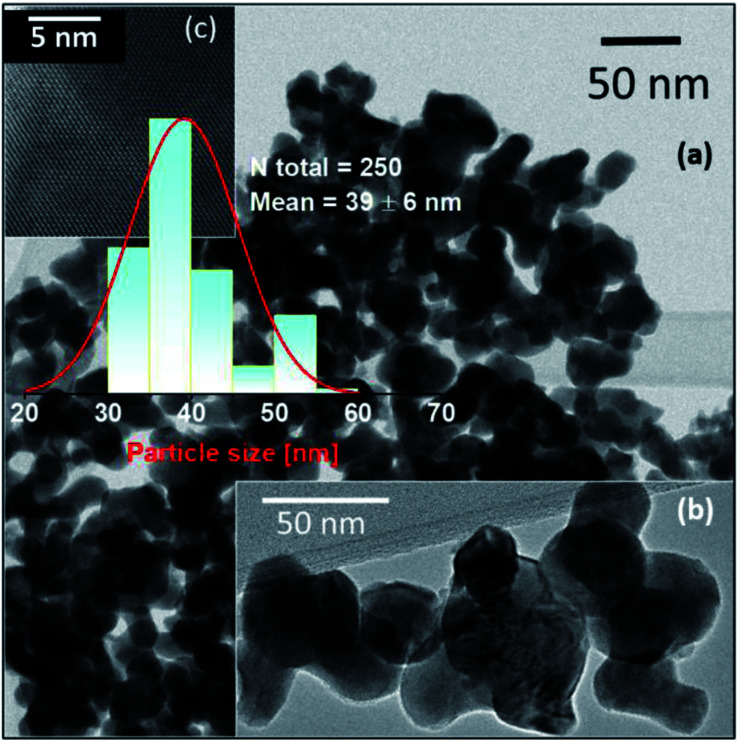
TEM images of 1 mol% Ba and 5 mol% Mn co-doped BFO (1BBFM) NPs at different regions are shown in (a) and (b) with the particle size distribution histogram. Image (c) shows a high-resolution TEM image at 5 nm scale bar for 1BBFM.

According to the XRD patterns, all NPs exhibit rhombohedral (*R*3*c*) crystal structure without any secondary phase above the XRD detection limit (Fig. 1). The unit cell volume and lattice parameters including microstrain of the same NPs have been reported in our earlier work.^14^ The Rietveld refinement analyses show distortion in the lattice parameters and an increased microstrain and decreased crystallite size upon Ba doping into Mn doped BFO NPs also in this study.^14^ In accordance with SEM measurements, both the pristine BFO and co-doped NPs manifest nearly spherical shape with an almost homogeneous size distribution (Fig. 2a–d). We have observed a decrement in the particle size upon co-doping which matches with previously reported PFM and TEM data, where the particle size decreases with a slight alteration in the morphology of the NPs.^14^ In Fig. 3a, b representative TEM images of the 1BBFM NPs are shown, and an average particle size of ∼39 nm is calculated. The high crystallinity of the 1BBFM NPs can be seen in Fig. 3c.

**Fig. 4:**
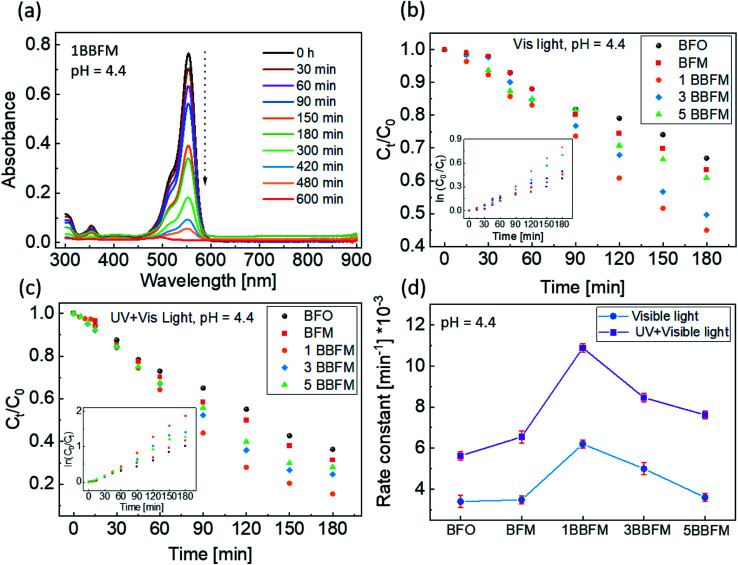
Absorption spectra of RhB with respect to time in the presence of 1BBFM NPs under visible light and at normal pH of the RhB solution (pH = 4.4) show the degradation of the RhB dye in (a). The relative concentration of RhB *versus* time under visible light and UV + visible light (at pH = 4.4) is shown in (b) and (c), respectively. The insets show the kinetics of the photodegradation. (d) Variation of the rate constants (*k*) for the photocatalysts under visible and UV + visible light excitation at pH = 4.4.

These co-doped BFO NPs were used to perform photodegradation of the organic dye RhB. The representative result of photodegradation of RhB using the 1BBFM sample is shown in Fig. 4a. The absorbance peak at 550 nm is a characteristic absorption feature of the RhB dye. A decrease in the magnitude of this peak with time indicates a decrease in the concentration of the dye in the solution due to photodegradation, since the absorbance is directly proportional to the concentration of the dye.^43^Fig. 4b compares the photodegradation of the dye within 180 minutes for all NPs under visible light illumination only (halogen lamp, with 390 nm cut-off filter) at pH = 4.4 of the solution. The photodegradation efficiency of BFM is slightly better than that of BFO NPs. However, incorporating 1 mol% Ba into BFM increases the efficiency drastically. For larger barium content in the co-doped NPs, the degree of photodegradation slightly decreases, but the 5BBFM NPs still show better photocatalytic properties than both BFM and BFO NPs. The photodegradation trend under the UV + visible light source (halogen lamp, without 390 nm filter) at pH = 4.4 is shown in Fig. 4c, where we observe the same trend as in Fig. 4b but with a slightly faster degradation for all samples. This shows an effect of UV light together with visible light on the photocatalytic efficiency of the studied NPs, which is due to the increase in the number of photons in total.

To study the kinetics of PC, Langmuir–Hinshelwood fitting was used.^44^ If the degradation/reaction follows a rate law (eqn (1)) then the reaction has first order kinetics.^45^

1 *kt* = ln(*C*_0_/*C*_*t*_)

here, *k* is the rate constant of the reaction, *C*_0_ is the initial concentration of the dye in the dark, and *C*_*t*_ is the concentration of the dye at time *t* during the PC reaction under light. Inserts in Fig. 4b and c show the representative time dependencies of ln(*C*_0_/*C*_*t*_) for a photocatalytic measurement under visible light and UV + visible illumination up to only 180 min, respectively. From the line fits of ln(*C*_0_/*C*_*t*_) in the time interval from 0 h to 5 h the slope (rate constant) values were obtained. The rate constant values for the doped and undoped NPs in the presence of UV and UV-visible light are plotted in Fig. 4d. For the 1BBFM NPs the rate constant is maximal and almost twice as high as for the pristine BFO NPs. However, further doping with Ba decreases the rate constant of the photocatalytic reaction under both illumination conditions. In short, doping increases the PC efficiency of the BFO NPs, and the best photocatalyst among all NPs found is 1BBFM under natural condition, pH = 4.4.

**Fig. 5:**
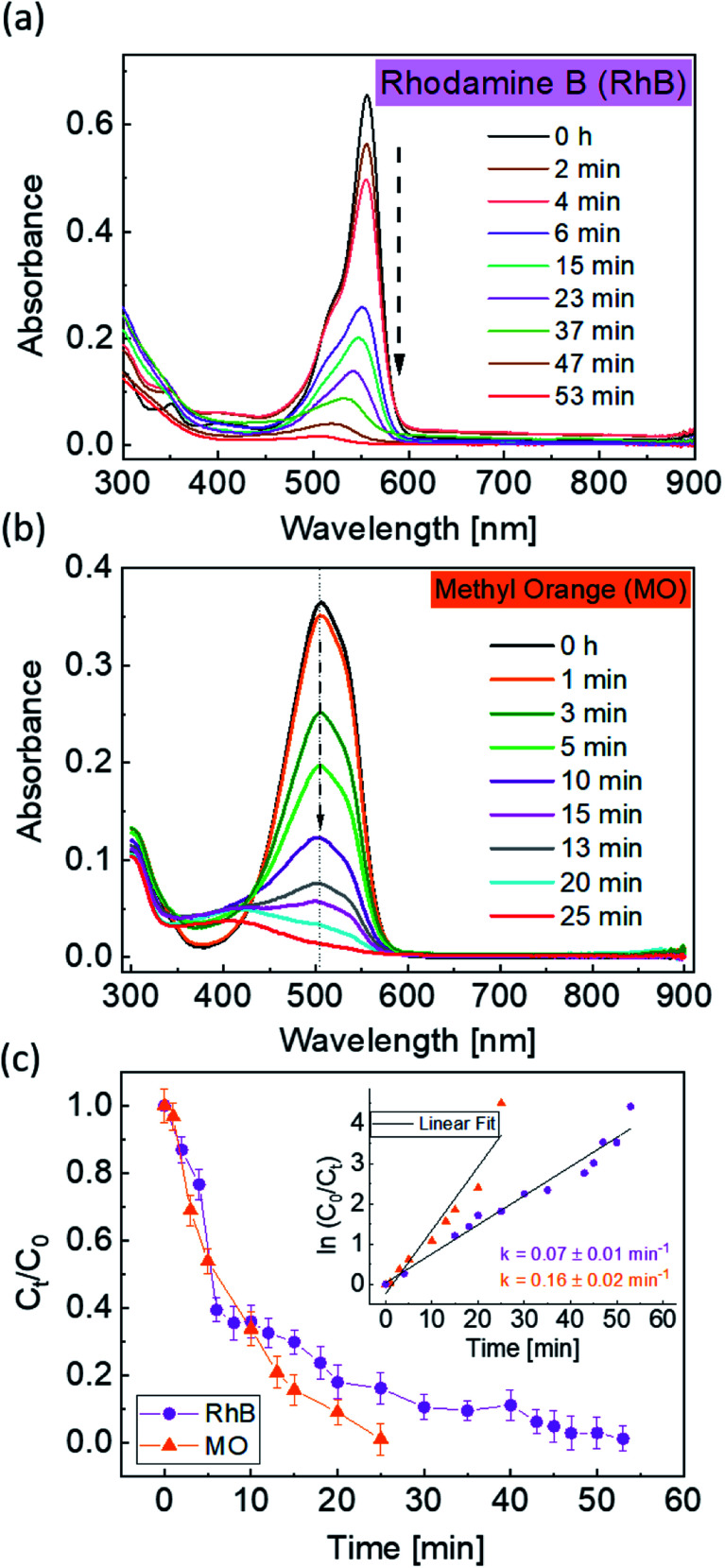
Absorbance plots with respect to time of RhB (a) and MO (b) in the presence of 1BBFM NPs at pH = 2.2 under UV + visible illumination. Photodegradation trend *C*_*t*_/*C*_0_ as a function of time for both dyes (c), where the inset shows the reaction kinetics and rate constant (*k*) values.

To further enhance the photocatalytic response of the 1BBFM NPs, we decreased the pH of the dye solution to 2.2 by adding a few drops of dilute HNO_3_. For such pH conditions and under UV-visible illumination, 1BBFM completely degraded the dye within 60 minutes (Fig. 5a). We also performed photodegradation of another organic dye, namely methyl orange under the same conditions. The 1BBFM NPs could completely degrade methyl orange within 25 minutes (Fig. 5b). From Fig. 5c, the photodegradation reaction follows first order kinetics for both dyes. The rate constant for RhB photodegradation increased by about 6 times at pH = 2.2 as compared to the solution with pH = 4.4. We observed that the nanopowders are better dispersed in RhB at pH = 2.2 in comparison to the natural condition (pH = 4.4). The zeta potential value of the dispersion increases from 17.2 mV at pH = 4.4 to 39.4 mV at pH = 2.2. This promotes more dye adsorption onto the NPs surface, and it could be one of the major causes for the increase in PC efficiency upon decreasing the pH of the solution.

These PC reactions were run at least three times and were found to have similar efficiencies as the first cycle. A representative graph of cycles for 1BBFM is shown in Fig. S2.† The NPs were removed after the PC with the help of a magnet attached to the PC jacketed beaker. For the photocatalyst it is important that it does not self-degrade and remains chemically inert with respect to the dye and solution during the PC reaction. To check the stability of the NPs we have performed XRD measurements for all NPs after the PC and found that they are stable. In Fig. S3† the XRD patterns of BFO and 1BBFM NPs are shown after PC at pH 2.2 and at pH 4.4 along with TEM images of 1 BBFM sample after PC at pH 2.2. From these results, we conclude that there is no formation of any secondary phase or degradation of the NPs, indicating that the NPs recover as a photocatalyst after successful PC. Additionally, we have performed total organic count (TOC) measurements for 1 BBFM (Fig. S4†), using a Shimadzu-TOC L device. From the TOC results, we found that all of the RhB was successfully degraded and decayed into CO_2_ and H_2_O.

Based on these data, the 1BBFM NPs shows the best photodegradation ability. What could be the reason of 1BBFM to be the best photocatalyst among the NPs under study? How does the overall doping into BFO NPs affect the photocatalysis? To unveil the possible reasons, we conducted further experiments to study the various parameters crucial for efficient photocatalysis in these samples. Photocatalysis involves the generation of charge carriers (e^−^ and h^+^) upon light absorption by the photocatalyst (see also Fig. S1†). Afterwards, the charge carriers get separated to diffuse from the bulk to the surface of the NPs along with competition against their recombination or trapping. In the last step, redox reactions occur at the surface of the NPs driven by the photogenerated charge carriers. Charge carriers not only promote dye reduction, but possibly also react with electron scavengers (*e.g.*, O_2_) to form radical anions (*e.g.*, O_2_^−^), oxidize organic molecules, or react with OH^−^/H_2_O to form ·OH radicals.^46^ So in the next sections, we will discuss about the light absorption by NPs and charge carrier generation, charge carrier recombination and available surface area for the photocatalysis process.

### Light absorption and charge carrier generation

To study the charge carrier generation by light absorption, we have performed UV-visible absorption measurements. There are different methods to collect absorption spectra of nanopowders. To avoid method-related ambiguities in the analysis of the data, we have first compared the absorption spectra of the pure BFO NPs using three methods. Fig. S5† shows the absorbance spectra obtained using the powder DRS method represented by the black line (a), the transmittance of the NPs dispersed in ethanol (blue line) (b), and the transmittance of the drop-cast and dried NPs (red line) (c). The spectra show a weak absorption feature at around ∼650 nm (1.90 eV) followed by a steep absorption edge at ∼550 nm (2.25 eV), possibly containing contributions from two different transitions. These results match well with earlier reports for polycrystalline bulk BFO and different sized BFO NPs.^47,48^ We observed that the drop-cast (red curve) and powder DRS (black curve) methods give well resolved and nearly similar shapes of the spectra, but for the dispersion (blue curve) method we only see the most prominent features of the absorption spectrum, where other important peaks are hidden. Here, we learn the effect of the chosen method on the absorption spectra of BFO NPs. Since these NPs exhibit strong scattering, the peak ∼650 nm diminishes in the transmittance measurement of the dispersed NPs, which was conducted in front of an integrating sphere. The collection of reflectance measurements in the solid form inside an integrating sphere to compensate scattering is a reliable way to observe the absorption features of these NPs.

**Fig. 6:**
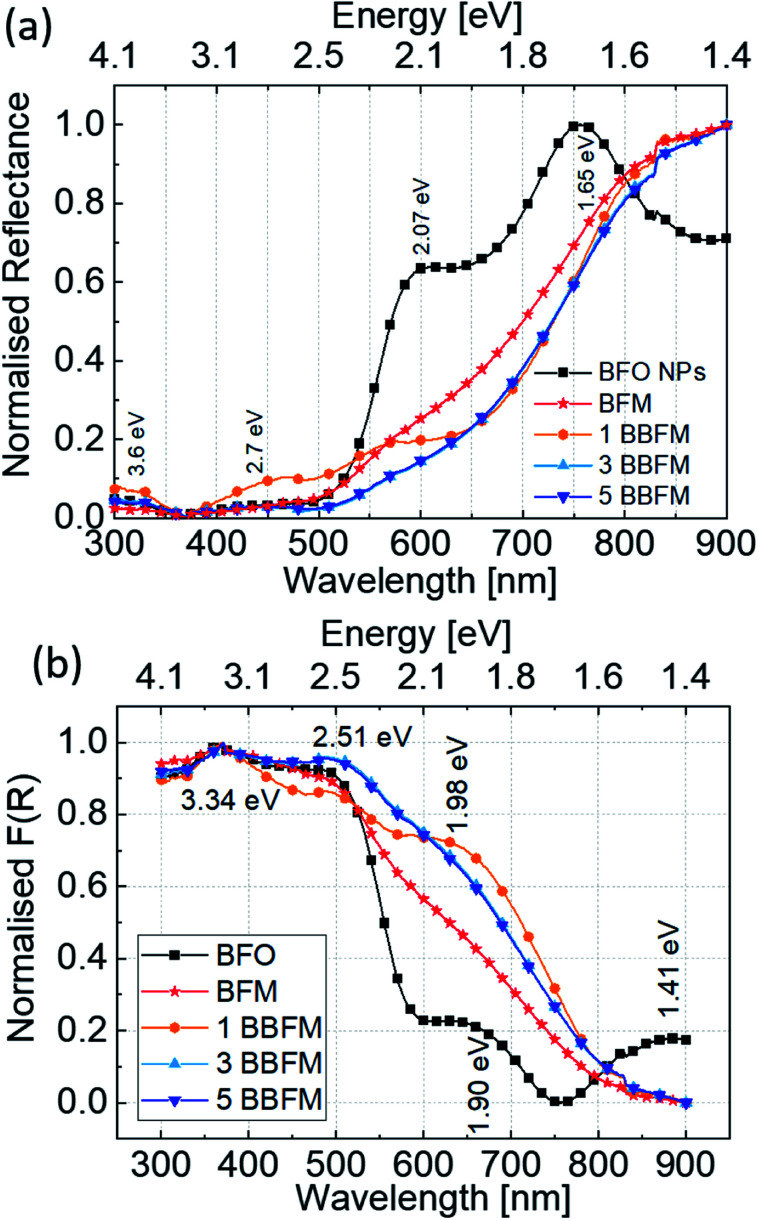
Normalised diffuse reflectance spectra (a) and Kubelka–Munk transformed (*F*(*R*)) normalised absorption spectra (b) for all Ba, Mn co-doped BFO NPs.

Based on the above experiment, we collected the powder DRS spectra of all synthesized nanopowders [Fig. 6a]. Fig. 6b shows the transformed absorption spectra of doped and undoped BFO NPs *via* the Kubelka–Munk function (*F*(*R*_∞_)).^49^

2 *F*(*R*_∞_) = *K*/*S* = (1 − *R*_∞_)^2^/*R*_∞_

3 *R*_∞_ = *R*_sample_/*R*_reference_

here, *R*_∞_ is the reflectance of an infinitely thick sample. *K* and *S* are the absorption and scattering coefficients.

The band gap of BFO is mainly formed by strong hybridization of Fe 3d, O 2p and Bi 6p orbitals,^50,51^ and it exhibits a complex electronic structure caused by spin-charge-lattice couplings due to the convolution of charge transfer bands (interatomic transitions) and absorption bands (d–d transitions).^47,52^ In Fig. 6b, the spectrum exhibits peaks at ∼1.41 eV (879 nm), ∼1.90 eV (652 nm), ∼2.50 eV (496 nm), and ∼3.34 eV (371 nm) in the limit of the measurement range. The peaks at lower energies, 1.41 eV and 1.90 eV, can be attributed to the crystal field d–d transitions ^6^A_1g_ → ^4^T_1g_ and ^6^A_1g_ → ^4^T_2g_ respectively.^48^ The peak at ∼2.50 eV may be attributed to the band gap of BFO NPs,^52^ and a hump near ∼3.34 eV may correspond to the well-known p → d charge transfer band.^53^ Upon Mn incorporation, the global shape of the BFO absorption spectra changes and upon further Ba doping the shape is altered again, which shows the individual effect of these dopants on the electronic structure of BFO NPs. The ^6^A_1g_ → ^4^T_2g_ transition (∼1.90 eV) becomes more intense and broader for BFM, which shows a distortion in energy levels due to the Jahn–Teller effect originating from the presence of Mn^3+^ ions as confirmed by XPS analysis in our earlier report.^14^ This peak is even more intense and shifted to a higher energy for 1BBFM. Upon further Ba doping, the absorption peak at ∼1.98 eV slightly broadens and the intensity of the peak ∼2.51 eV increases. One can see that in the photon energy range from 1.6 to 2.50 eV the Ba and Mn co-doped NPs absorb more solar light than the BFM and undoped BFO NPs.

In literature, BFO is often considered as direct band gap semiconductor by both theoretical and experimental proofs^54–56^ whereas there are also reports claiming an indirect band gap.^57–59^ The band gap is considered to be ∼2.5 eV for BFO single crystals.^55^ For BFO NPs, the band gap is extracted to be 2.74 eV for 100 nm, 2.24 eV for 190 nm, 2.22 eV for 120 nm, 2.21 eV for 50 nm, and 2.18 eV for 30 nm NPs using the Tauc model from optical methods.^48,60^ However, the Tauc plot model is based on a three-dimensional density of states and thus does not include, *e.g.*, d–d transitions. Therefore, we refrain from using the Tauc plot approach here for estimating the band gap for doped NPs. So far, we have observed the distortion in electronic structure of BFO, and increased light absorption by doping the NPs. The utilization of maximum solar light for charge carrier generation is reflected by the increased photocatalytic activity of the doped NPs.

### Local ferroelectric properties

**Fig. 7:**
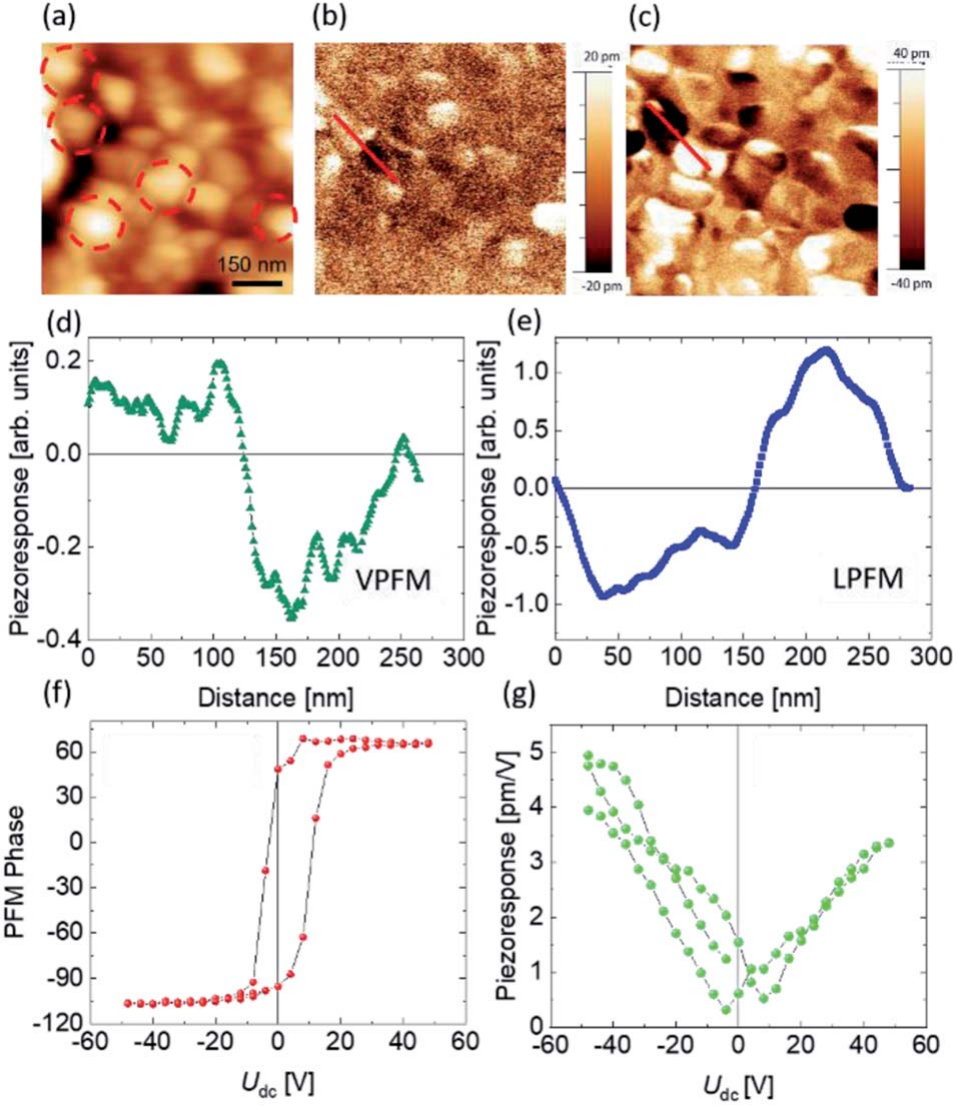
Topography (a), vertical (b) and lateral (c) PFM images of 1BBFM NPs. The cross sections of vertical PFM (d) and lateral PFM (e) images (the cross-section location is marked by a red line in the corresponding images). The local piezoresponse phase (f) and amplitude (g) hysteresis loops.

After charge carrier generation, their efficient separation also plays a crucial role during the photocatalysis process. In a previous report we have proven by PFM technique that BFO NPs and their co-doped fellows are ferroelectric.^14^ In ferroelectric materials, it has been shown that charge carriers get separated easily upon light illumination.^61^ In photoferroelectric materials a broad space charge region exists due to internal screening of spontaneous polarization by free charge carriers and defects within the material. The resulting band bending depends on the surface polarity and facilitates the separation of photogenerated charge carriers.^62,63^ One can expect that the larger spontaneous polarization of NPs will be beneficial for charge carrier separation and photocatalytic activity. To address the polarization of our best photocatalyst *i.e.*, 1BBFM NPs we applied PFM. The PFM signal depends on the local piezoresponse, which is proportional to the polarization value. Fig. 7 shows the topography (a), vertical PFM (VPFM) and lateral PFM (LPFM) images. The intensities of the VPFM and LPFM signals depend on the out-of-plane and in-plane components of polarization, respectively. The 1BBFM NPs are mostly single domain as shown by red dotted circles, similar to the BFO and BFM NPs.^14^ The local PFM hysteresis loops (Fig. 7f, g) confirm the switching of the polarization direction by an external electric field confirming the ferroelectric state of the NPs. In our previous study we found that Mn doping slightly increases the PFM signal of the BFO NPs, but co-doping with 5 mol% Ba decreases it.^14^ Comparing the PFM response of the 1BBFM NPs with those data, one can see that the intensity of the PFM is about 30% larger than in the BFM NPs (Fig. S6†). As piezoresponse of a material is proportional to the spontaneous polarization, we can conclude that 1BBFM has a larger spontaneous polarization than the BFO and BFM NPs. A larger polarization means a larger depolarization field, which can facilitate photoinduced charge carrier separation and may best explain the PC efficiency of the 1BBFM NPs to be the best.

### Charge carrier recombination

Once the charge carriers are separated, their recombination should be inhibited for their diffusion from the bulk to the surface of the NPs. The PL spectroscopy can be used to identify not only the defect states but the recombination of charge carriers as well.^64^ Therefore, the PL spectra of all NPs were measured by excitation of the NPs by a 450 nm (2.75 eV) laser light source (Fig. S7†). For both, the pristine and doped BFO NPs, we found similar emission peaks, which indicates that no new types of in-band defect levels are formed at this dopant concentration. However, the peaks get slightly shifted, but this effect is not as significant as the change in the total peak area (Fig. S7b†). The 1BBFM sample has the smallest peak area for both emission peaks among the NPs, which is associated with a lower charge carrier recombination,^65^ and a less absorption of 1BBFM at ∼500 nm (2.48 eV). Therefore, the larger PL peak area for the 5BBFM NPs than for the 1BBFM NPs may correlate with the lower PC efficiency of the 5BBFM sample.

### Available surface area for photocatalysis

**Fig. 8:**
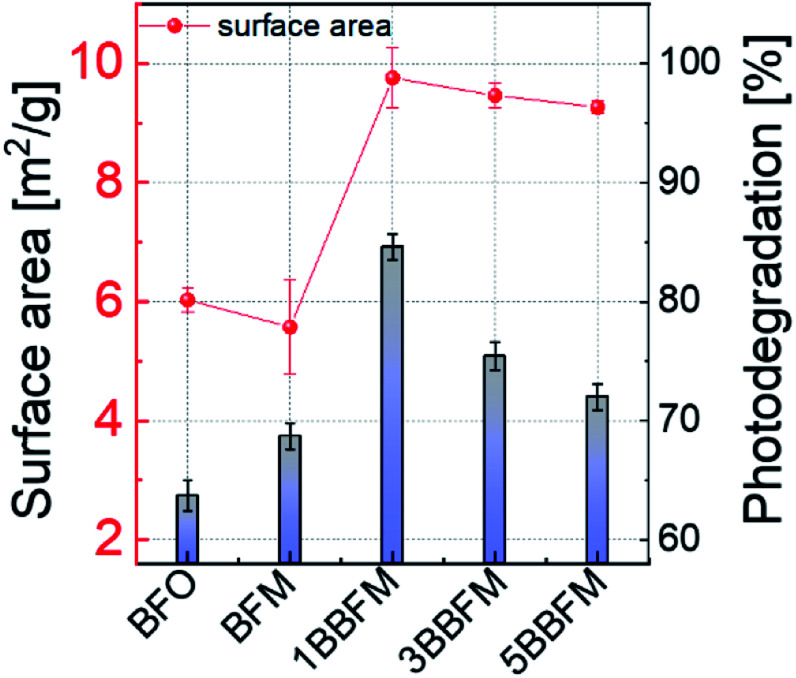
The specific surface area trend (red) *versus* doping in BFO NPs is compared to the relative photodegradation (vertical bars) of RhB under UV-visible light (pH = 4.4) up to 180 minutes.

The specific surface area of the co-doped BFO NPs was evaluated using BET measurements. The results are summarized in Table S1.† Doping with Mn alone does not lead to a significant change in the surface area, but by adding 1% Ba the surface area increases considerably. For the samples with more Ba content, the surface area decreases slightly, but they still exhibit more surface area than the pure BFO NPs. The trend of the specific surface area compared to the photodegradation trend at pH = 4.4 under UV-visible light for all NPs is shown in Fig. 8.

**Fig. 9:**
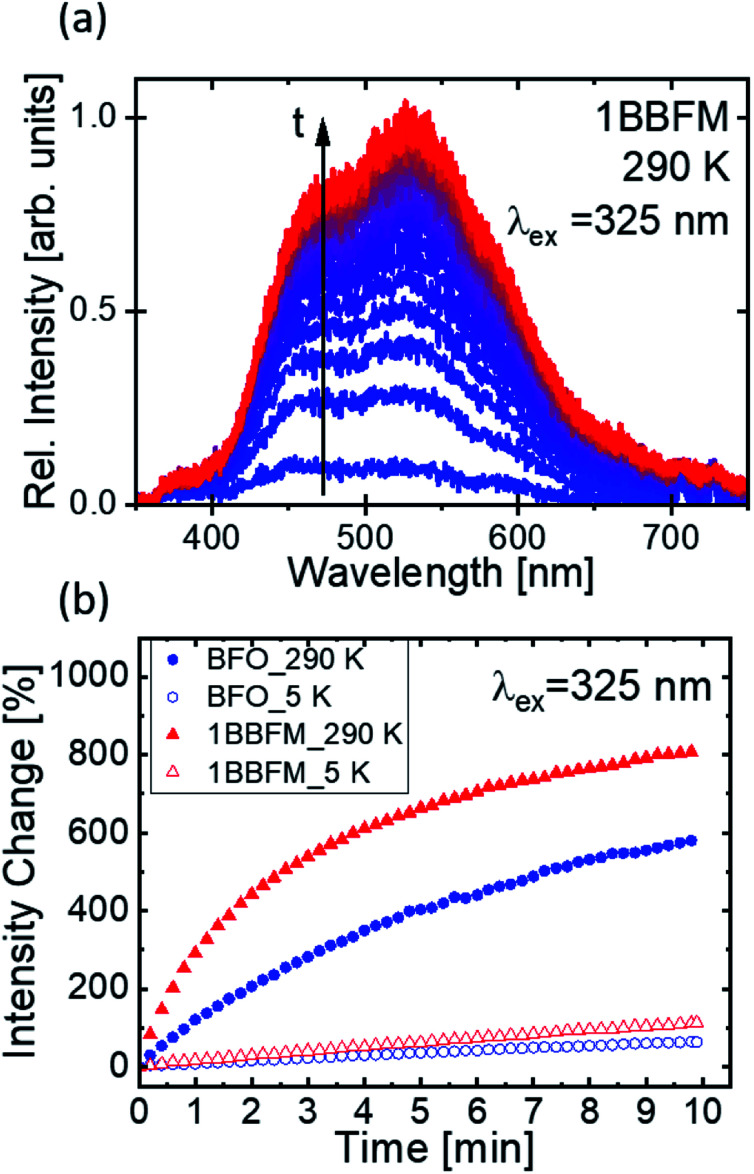
(a) The illumination time (*t*) dependent emission spectra of 1BBFM NPs at 290 K with 325 nm excitation. (b) The change in intensity *versus* illumination time in minutes at 290 K and 5 K for pristine BFO NPs (blue) and 1BBFM NPs (red), respectively.

Though surface area can be a major factor, other factors also affect the degradation rate of doped NPs. As photocatalysis is a surface-mediated process, not only the available surface area of the NPs plays an important role for their efficiency, but also the surface activity. It has been demonstrated that for a variety of metal-oxides, visible fluorescence is connected to the adsorption and modification of surface adsorbates such as oxygen or -OH_x_ groups by the excitation light.^68,69^ The evolution of such fluorescence under continuous excitation is hence connected to the surface activity. To support the beneficial properties of our co-doped NPs as compared to pure BFO, we drop cast a dispersion of the NPs in ethanol onto a cleaned Si substrate and conducted PL measurements under UV excitation. The dried NPs were excited with a 325 nm (3.81 eV) laser source in vacuum to collect PL spectra at 290 K and at 5 K. We find the PL spectra in the visible range from 400–700 nm. During continuous illumination, a strong variation of the PL intensity of all samples was observed as exemplified by the spectra of the doped 1BBFM NPs (Fig. 9a). No change can be detected in the shape of the spectra.

The occurrence of such broad spectra, as well as the observed change in intensity, has been described in literature and is most likely associated with the adsorption of −OH_*x*_ groups from the solvent (or atmosphere) onto the NPs surface and their modification under the action of incident laser radiation.^66,67^ Although the experiments were conducted under high vacuum, the –OH groups are expected to still persist on the highly porous NPs surfaces and to be trapped between agglomerated particles.

The observed brightening of the PL spectra in the course of illumination manifests light induced surface activity attributed to the modification of adsorbed OH-groups on the surface of the NPs. Comparing the emission intensity change of the pristine and 1BBFM NPs (Fig. 9b) reveals a much stronger brightening effect for the 1BBFM sample at both room and cryogenic temperatures. This effect indicates a higher optically induced surface activity of 1BBFM in terms of the adsorption and alteration of attached –OH groups as compared to the pristine BFO NPs. The improved adsorption of –OH groups will lead to increased ·OH radical formation after charge carrier generation. This effect is expected to improve the photocatalytic efficiency of the doped sample in the presence of a dye.

## Discussion

The obtained results indicate that the increased photocatalytic efficiency of BFO NPs upon Ba and Mn doping can be explained by a combination of several factors, where the foremost is the alteration in the lattice parameters and the change in size and morphology of the NPs. The spherical morphology of the doped BFO NPs under study implies a large surface area, which provides more sites for incoming charge carriers to participate in redox reactions at the surface. The 1BBFM sample has the largest surface area of almost ∼1.6 times more than that of the pure BFO NPs (Fig. 8). The larger surface area and the higher pore volume are major factors in the photocatalytic properties of the co-doped BFO NPs.

Second, the optical absorption edge in the electronic structure of the BFO NPs is altered due to doping. In the Mn doped BFO NPs, the existence of manganese both as Mn^3+^ (Jahn–Teller cation) and Mn^4+^ in the crystalline lattice was confirmed by XPS and Raman analyses.^14^ The presence of a Jahn–Teller cation in the MnO_6_ octahedra can distort the electronic structure of BFO by splitting the energy levels further.^68^ Upon further co-doping Ba into BFM, the unit cell volume increases and the Mn^3+^ concentration reduces in favor of Mn^4+^. It is important to notice that this effect is most significant for the 1BBFM, which also has the largest unit cell volume.^14^ For co-doped BFO NPs, two main factors cause important changes in the electronic structure: increased unit cell volume due to bigger Ba^2+^, and presence of a Jahn–Teller cation (Mn^3+^). In total, such structural distortion leads to an alteration in Fe–O bond lengths and influences the d–d crystal field transition energy levels,^68^ and can alter the electronic transition of *C*_3v_ crystal symmetry of BFO. As per Wei *et al.*, an increase in crystal field splitting due to the reduction in the unit cell volume *via* doping by rare-earth elements leads to a decrease in the energy edge of d–d transitions.^69^ In our case the increase in unit cell volume due to Ba doping into BFM leads to an increase of the ^6^A_1g_ → ^4^T_2g_ d–d transition energies (Fig. 6b). The increase of the light absorption associated with the ^6^A_1g_ → ^4^T_2g_ d–d transition due to the doping influences the photocatalytic efficiency significantly.

Third, the spontaneous polarization of these ferroelectric NPs at room temperature causes band bending, which assists the separation of charge carriers. This effect can also promote the photocatalysis process considerably. The PFM data indicate the larger polarization in the 1BBFM NPs as compared to other NPs under study, which correlates with their best photocatalytic performance.

The fourth factor is the slowest charge carrier recombination for the 1BBFM among all the NPs, as evidenced by the decreased peak area of the PL emission spectra collected at 450 nm.

The temporal evolution of the PL intensities collected at 325 nm provides a conclusive link to the catalytic activities of the NPs *via* adsorbing –OH groups onto their surface. The increasing rate of the PL intensity growth under continuous illumination observed in the 1BBFM sample as compared to the undoped BFO indicates stronger interaction of the nanoparticle surface with adsorbed OH groups. This finding also relates the increased photocatalytic efficiency of the 1BBFM NPs to the increased surface activity.

## Conclusions

Ba and Mn co-doped BFO NPs synthesized by a modified sol–gel method show enhanced photocatalytic activity for the degradation of organic dyes (rhodamine B and methyl orange) under UV-Visible light. Among them the NPs with 5 mol% Mn and 1 mol% Ba doping show the best results and can degrade MO and RhB dyes in 25 and 60 minutes, respectively, at pH = 2.2. The surface area and pore volume increase for the doped NPs, while the 1BBFM NPs have the largest surface area and pore volume amidst the NPs under study. The electronic band structure of BFO gets altered due to doping. This increases the light absorption capability of doped NPs in the visible range, in particular between 500 and 800 nm. The larger spontaneous polarization and related depolarization field make the charge carrier separation more effective in the 1BBFM NPs than in the BFO and other doped NPs. The emission spectra show that for 1BBFM the charge carrier recombination is lower than for other NPs. A stronger increase of luminescence from adsorbed species on the co-doped NPs during continuous UV excitation compared to the pristine BFO NPs further indicates an enhanced surface activity. Overall, the maximum photocatalytic efficiency for the 1BBFM NPs is attributed to the cooperative effect of increased light absorption, a larger surface area, more effective charge separation, and less recombination of photogenerated charge carriers. The dopant concentration is a key factor in controlling the parameters of the photocatalytic process. In our case doping with 1 mol% Ba into BiFe_0.95_Mn_0.05_O_3_ NPs is proven to be optimal for the photocatalysis efficiency.

## Conflicts of interest

The authors declare no conflict of interest.

## Author contributions

A D has synthesized the NPs and performed structural, morphological, reflectance and emission characterization of NPs *via* XRD, TEM, SEM, UV-Vis, and PL methods. A D performed all photocatalysis experiments and wrote the manuscript. V V S performed the PFM measurements and analyzed with A D. A S has collected the PL measurements with respect to illumination time and analyzed the results with A D. M E C has initialized this research and was responsible for scientific project administration. V V S, G B and D C L have contributed equally to the scientific discussion.

## Supplementary Material

NA-003-D1NA00420D-s001
