# Supplementary material for: Role of cooperative factors in the photocatalytic activity of Ba and Mn doped BiFeO3 nanoparticles
Source: Nanoscale Adv. 2021 Aug 26;3(20):5830–40. doi: 10.1039/d1na00420d (PMC9419078; doi:10.1039/d1na00420d)
Supplement: NA-003-D1NA00420D-s001 [file NA-003-D1NA00420D-s001.pdf]

Electronic Supplementary Material (ESI) for Nanoscale Advances.
This journal is © The Royal Society of Chemistry 2021

Supplementary Information.

Role of Cooperative Factors in the Accelerated Photocatalytic Activity of Ba and Mn Doped $BiFeO_3$ Nanoparticles

Astita Dubey,[a] Alexander Schmitz,[b] Vladimir V. Shvartsman,[a] Gerd Bacher,[b] Doru C. Lupascu,[a] Marianela Escobar Castillo[a]

[a] *Institute for Materials Science and Center for Nanointegration Duisburg-Essen (CENIDE), University of Duisburg-Essen, 45141, Essen, Germany.*
[b] *Werkstoffe der Elektrotechnik & CENIDE, Universität Duisburg-Essen, Bismarckstraße 81, 47057 Duisburg, Germany.*

List of figures and tables.

Figure S1: Schematic shows the major steps involved in the photocatalysis process. Vertical lines show the (i) charge carrier generation, (ii) recombination, and (iii) trapping.

Figure S2: Recyclability of 1BBFM co-doped BFO NPs in the degradation of methyl orange (a) and of RhB (b).

Figure S3: XRD diffractograms of photocatalysts before and after photocatalytic (PC) cycles shown in (a) and (b) for 1 mol % Ba 5 mol % Mn co-doped BFO NPs (1BBFM) and BFO NPs, respectively. (i) before photocatalysis, (ii) after 3 PC cycles at pH 4.4 and (iii) shows after PC cycle at pH 2.2 for NPs. The XRD diffractograms, of the NPs do not change after PC cycles in both pH conditions, indicating that the NPs are stable, functional and reusable for further photocatalysis. In right, (c), and (d) show TEM images of 1BBFM after PC at pH 2.2.

Figure S4: Total organic count (TOC) values after photocatalysis at pH 2.2 for 1 mol % Ba 5 mol % Mn BFO NPs (1BBFM) photocatalysts. The TOC value is also recorded after 24 h (1440 min), and it matches with total photodegradation time ~60 min.

Figure S5: Absorbance spectra of BFO NPs obtained by different methods, where the black curve shows transformed reflectance of pressed nanopowders, the blue curve shows absorption spectrum of BFO NPs dispersed in ethanol, and the red one shows transmittance of drop casted BFO NPs on a quartz substrate.

Figure S6: Comparison between cross-sections of the PFM signal normalized to the driving voltage for the BFO, BMF, and 1BBMF NPs. The data for the BFO and BMF NPs are taken from reference 1.

Figure S7: The emission spectra of doped and undoped BFO NPs at 298 K excited by a 450 nm laser source shown in (a), the peak area trend of the peaks at ~572 nm and ~613 nm is shown for all NPs in (b).

Table S1: The specific surface area values and pore volume values for all co-doped BFO NPs.

Reference.

1. A. Dubey, M. Escobar Castillo, J. Landers, S. Salamon, H. Wende, U. Hagemann, P. Gemeiner, B. Dkhil, V. V. Shvartsman and D. C. Lupascu, J. Phys. Chem. C, 2020, 124, 22266–22277.

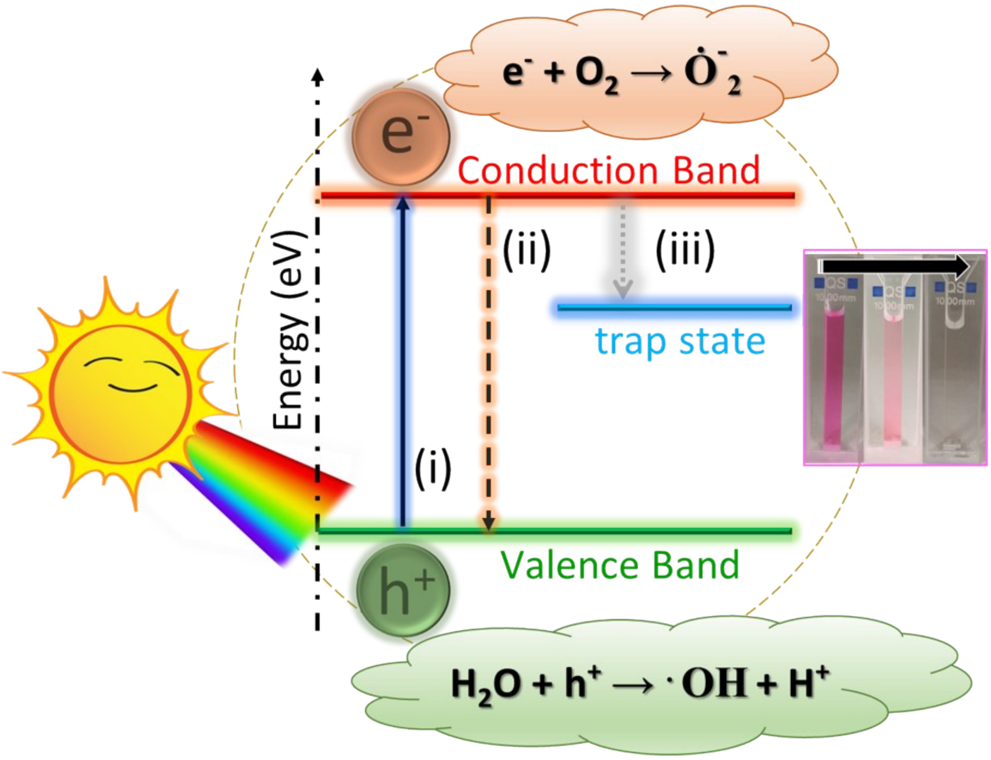

Figure S1: Schematic shows the major steps involved in the photocatalysis process. Vertical lines show the (i) charge carrier generation, (ii) recombination, and (iii) trapping.

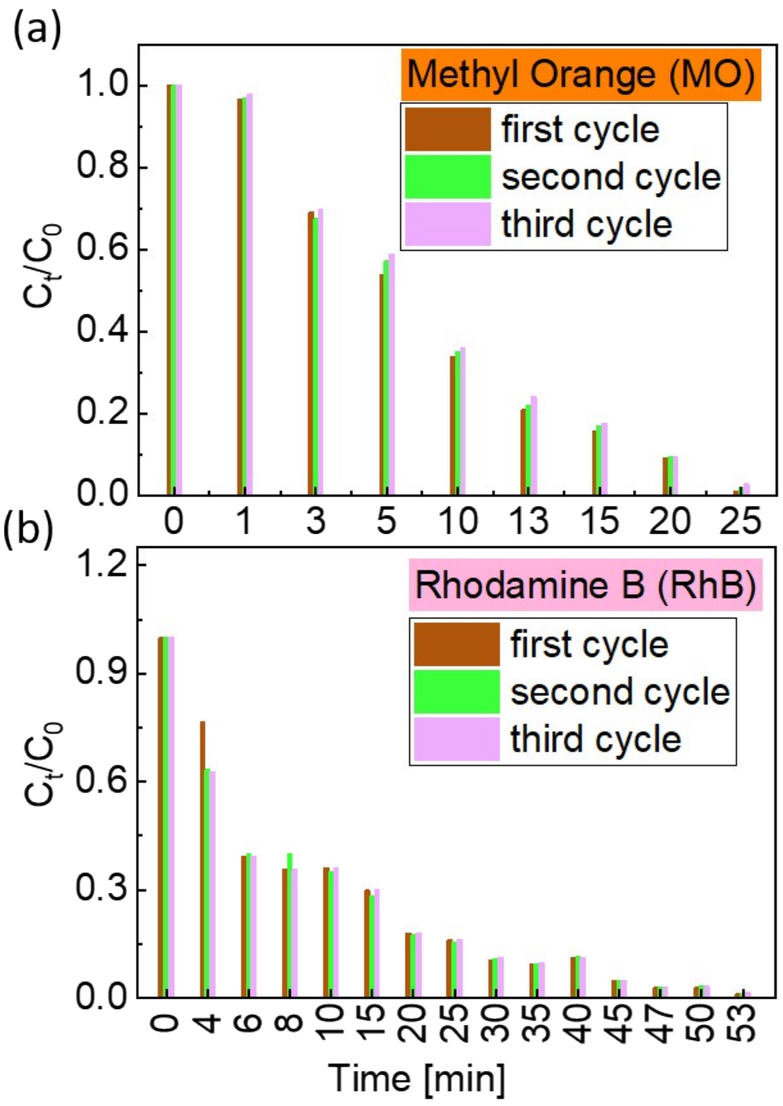

Figure S2: Recyclability of 1BBFM co-doped BFO NPs in the degradation of methyl orange (a) and of RhB (b), where C_t/C_o is the photodegradation of the dye.

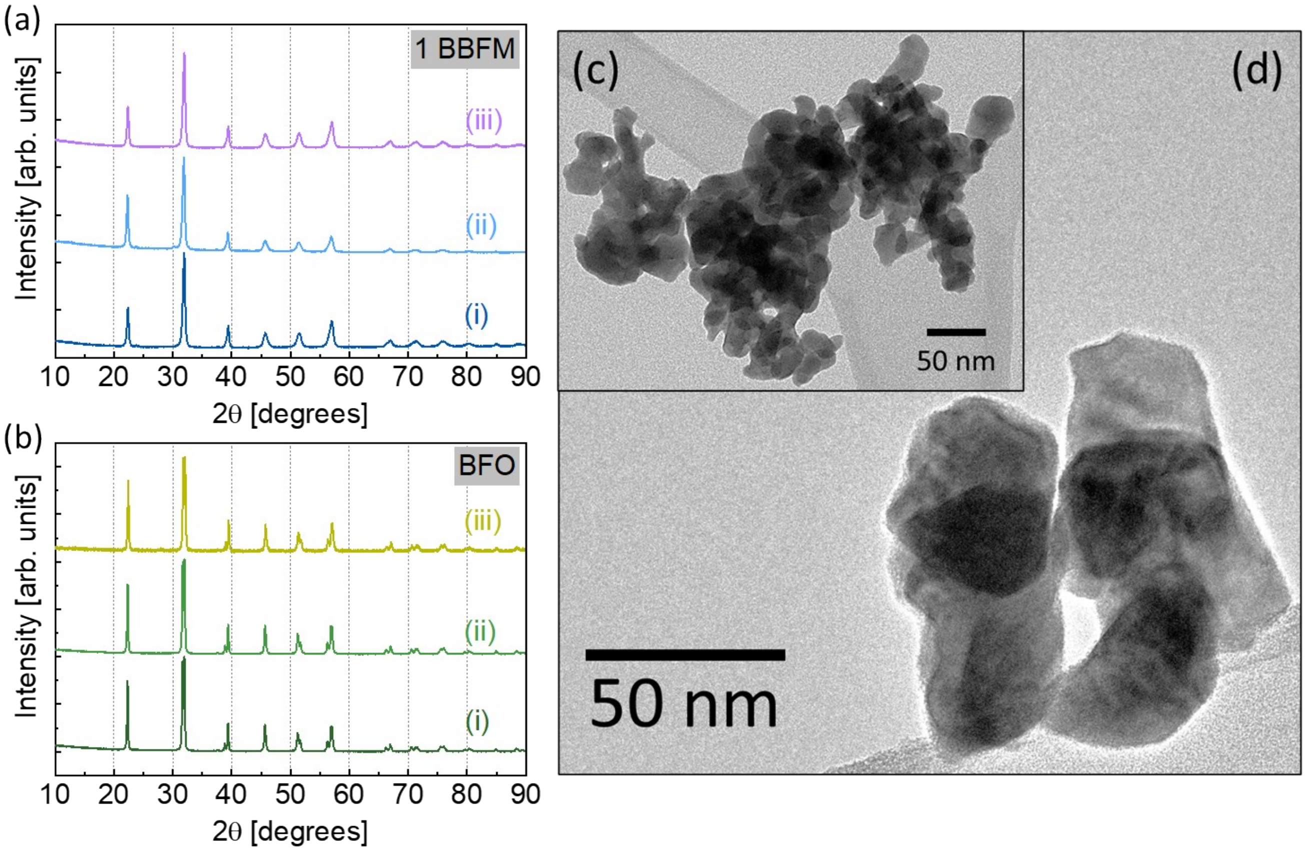

Figure S3: XRD diffractograms of photocatalysts before and after photocatalytic (PC) cycles shown in (a) and (b) for 1 mol % Ba 5 mol % Mn co-doped BFO NPs (1BBFM) and BFO NPs, respectively: (i) before photocatalysis, (ii) after 3 PC cycles at pH 4.4 and (iii) after one PC cycle at pH 2.2. The XRD diffractograms, of the NPs do not change after PC cycles at both pH conditions, indicating that the NPs are stable, functional, and reusable for further photocatalysis. On the right, (c), and (d) show TEM images of 1BBFM after PC at pH 2.2.

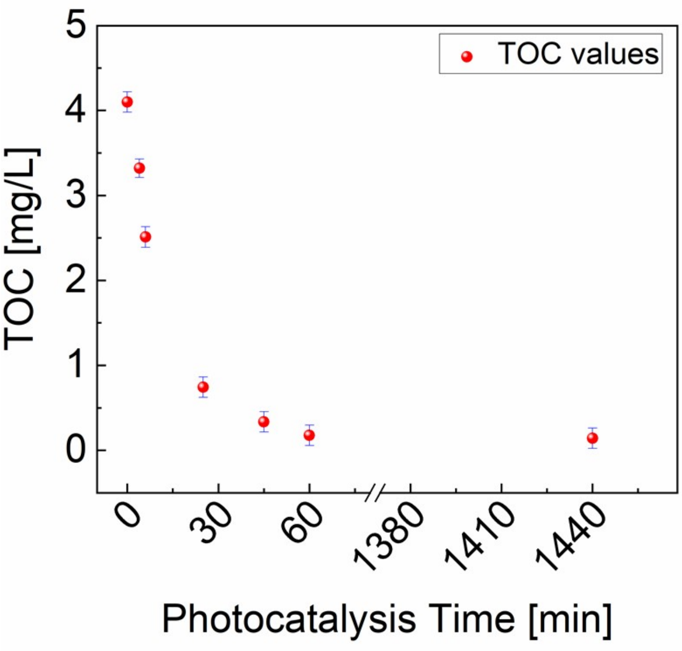

Figure S4: Total organic count (TOC) values after photocatalysis at pH 2.2 for 1BBFM photocatalysts. The TOC value was also recorded after 24 h (1440 min), and it matches with the total photodegradation time ~60 min.

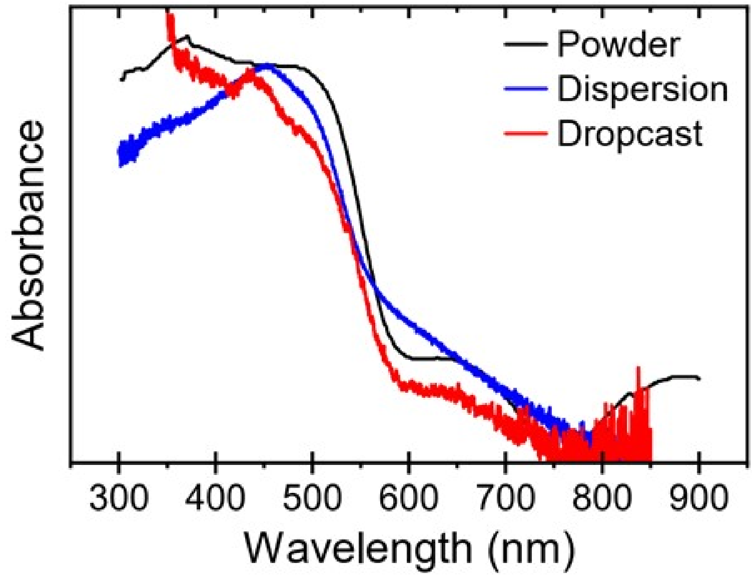

Figure S5: Absorbance spectra of BFO NPs obtained by different methods, where the black curve shows transformed reflectance of pressed nanopowders, the blue curve the absorption spectrum of BFO NPs dispersed in ethanol, and the red one the absorption of drop cast BFO NPs on a quartz substrate. Here, the blue and red curves were measured in front of an

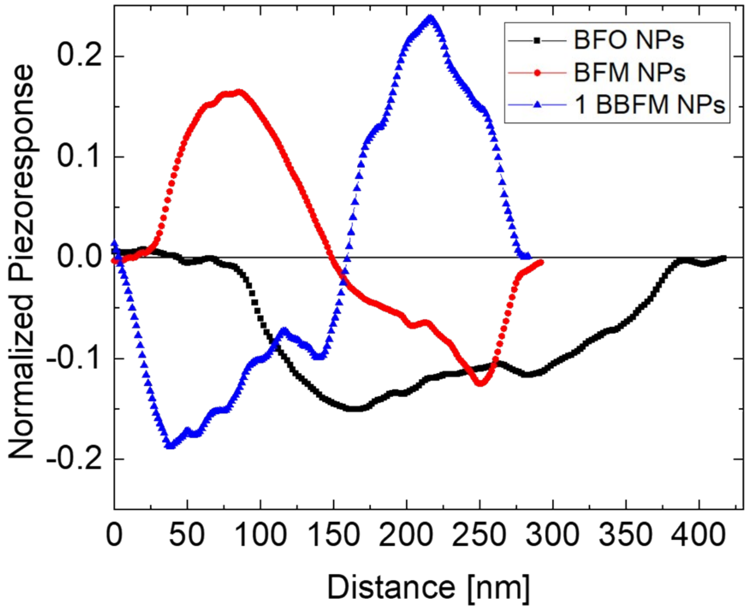

Figure S6: Comparison between cross-sections of the PFM signal normalized to the driving voltage for the BFO, BMF, and 1BBMF NPs. The data for the BFO and BFM NPs are taken from ***reference 1.***

integrating sphere in transmission geometry.

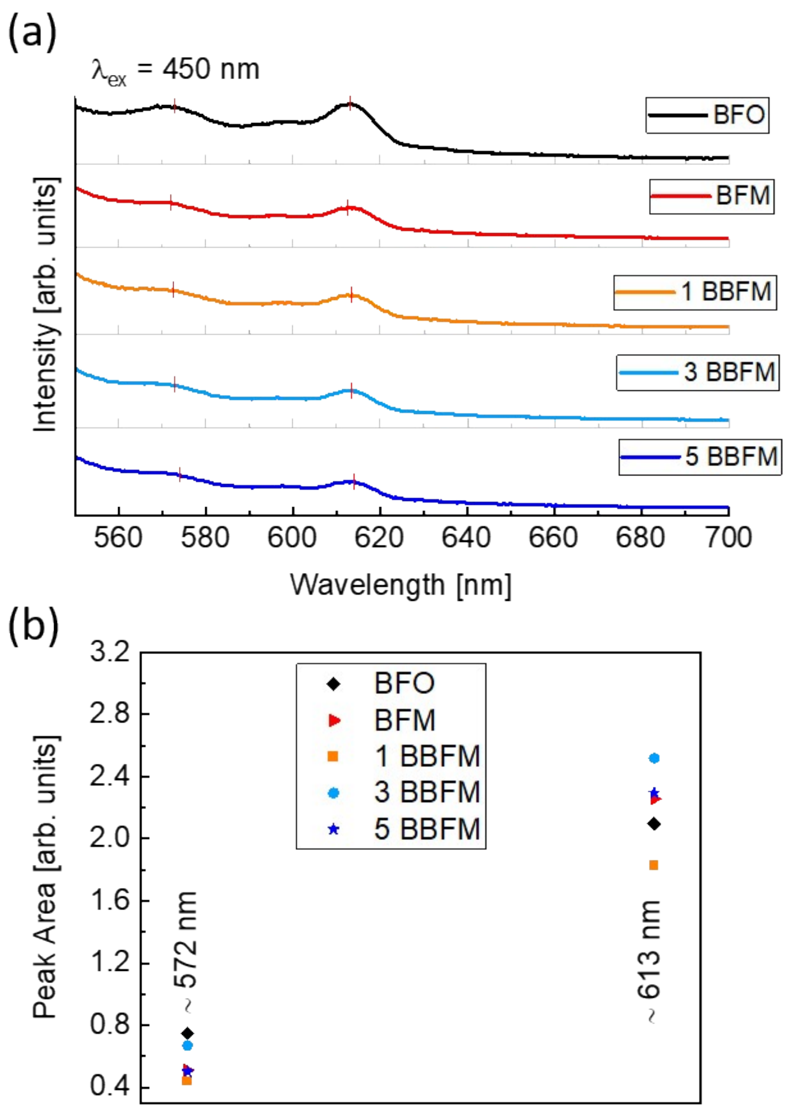

Figure S7: The emission spectra of doped and undoped BFO NPs at 298 K excited by a 450 nm laser source shown in (a), the peak area trend of the peaks at ~ 572 nm and ~ 613 nm is shown for all NPs in (b).

Table S1: The specific surface area and pore volume values for all co-doped BFO NPs.

**Elements**	**BFO**	**BFM**	**1 BBFM**	**3 BBFM**	**5 BBFM**
**Surface Area (m^2/g)**	6.0 ± 0.2	5.57 ± 0.08	9.76 ± 0.05	9.47 ± 0.02	9.27 ± 0.04
**Pore Volume (ml/g)**	0.032 ± 0.004	0.030 ± 0.003	0.067 ± 0.005	0.052 ± 0.001	0.049 ± 0.006
